# Elucidation of the Mechanism of Host NMD Suppression by HTLV-1 Rex: Dissection of Rex to Identify the NMD Inhibitory Domain

**DOI:** 10.3390/v14020344

**Published:** 2022-02-09

**Authors:** Kazumi Nakano, Nobuaki Karasawa, Masaaki Hashizume, Yuetsu Tanaka, Takeo Ohsugi, Kaoru Uchimaru, Toshiki Watanabe

**Affiliations:** 1 Department of Computational Biology and Medical Sciences, Graduate School of Frontier Sciences, The University of Tokyo, Tokyo 108-8639, Japan; 2 Faculty of Medicine, University of the Ryukyus, Nishihara 903-0125, Japan; 3 Department of Laboratory Animal Science, School of Veterinary Medicine, Rakuno Gakuen University, Ebetsu 069-8501, Japan; 4 Department of Practical Management of Medical Information, Graduate School of Medicine, St. Marianna University, Kawasaki 216-8511, Japan

**Keywords:** HTLV-1 rex, NMD inhibition, viral RNA, Upf1, Upf3, SMG5/7

## Abstract

The human retrovirus human T-cell leukemia virus type I (HTLV-1) infects human T cells by vertical transmission from mother to child through breast milk or horizontal transmission through blood transfusion or sexual contact. Approximately 5% of infected individuals develop adult T-cell leukemia/lymphoma (ATL) with a poor prognosis, while 95% of infected individuals remain asymptomatic for the rest of their lives, during which time the infected cells maintain a stable immortalized latent state in the body. It is not known why such a long latent state is maintained. We hypothesize that the role of functional proteins of HTLV-1 during early infection influences the phenotype of infected cells in latency. In eukaryotic cells, a mRNA quality control mechanism called nonsense-mediated mRNA decay (NMD) functions not only to eliminate abnormal mRNAs with nonsense codons but also to target virus-derived RNAs. We have reported that HTLV-1 genomic RNA is a potential target of NMD, and that Rex suppresses NMD and stabilizes viral RNA against it. In this study, we aimed to elucidate the molecular mechanism of NMD suppression by Rex using various Rex mutant proteins. We found that region X (aa20–57) of Rex, the function of which has not been clarified, is required for NMD repression. We showed that Rex binds to Upf1, which is the host key regulator to detect abnormal mRNA and initiate NMD, through this region. Rex also interacts with SMG5 and SMG7, which play essential roles for the completion of the NMD pathway. Moreover, Rex selectively binds to Upf3B, which is involved in the normal NMD complex, and replaces it with a less active form, Upf3A, to reduce NMD activity. These results revealed that Rex invades the NMD cascade from its initiation to completion and suppresses host NMD activity to protect the viral genomic mRNA.

## 1. Introduction

In the period 1980–1981, human T-cell leukemia virus type I (HTLV-1) was discovered as a human retrovirus in Japan and the USA, and was later shown to be the causative agent of adult T-cell leukemia/lymphoma (ATL) [1,2,3,4,5]. HTLV-1 mainly targets CD4^+^ T cells and causes ATL in approximately 5% of infected individuals after an incubation period of 50–60 years [6]. In contrast, 95% of infected individuals remain asymptomatic throughout their lives, and infected cells maintain a stable immortalized latent state in the body for decades [7]. We hypothesize that the function of HTLV-1 viral accessory proteins from infection to transition to latency influences the phenotype of infected cells during latent infection.

HTLV-1 is a sense-stranded RNA virus belonging to the genus *Deltaretrovirus* in the family *Retroviridae*. After infection, the virus undergoes reverse transcription and is permanently integrated into the human genomic DNA of the host cell as a 9 kb provirus with a long terminal repeat (LTR) at both ends. The provirus first transcribes a fully (twice) spliced *Tax/Rex* mRNA. Tax strongly activates the LTR, promotes transcription of viral genes, and disrupts various gene expression, signal transduction, cell cycle regulation, and DNA damage responses in the host cell. It is thought to be involved in the immortalization and transformation of HTLV-1-infected T cells [7,8,9,10,11,12]. Rex binds specifically to the Rex recognition element (RxRE) in the 3′-UTR of HTLV-1 mRNA and mediates nuclear export of viral mRNA in a CRM1-dependent manner [13,14]. In particular, Rex facilitates the extranuclear trafficking of unstable non-spliced *Gag/Pro/Pol* mRNA and incompletely spliced *Env* mRNA to promote the production of viral particles [15,16,17,18,19]. This is accompanied by a decrease in the level of Tax/Rex mRNA expression and a shift to a latent infection state. Rex is therefore thought to regulate the timing of HTLV-1 replication and latency [20,21,22].

Rex is a 27 kDa phosphoprotein consisting of 189 amino acids and has seven phosphorylation sites: Thr-22, Ser-36, Thr-37, Ser-70, Ser-97, Ser-106 and Thr-174, of which Ser-97 and Thr-174 are thought to be important in the transport of viral mRNA [23]. Rex has several domains, which are nuclear/nucleolus localization signals (NLS) for importin-β-mediated nuclear translocation and arginine-rich motifs (ARM) for RNA binding via the RxRE (aa1–19); the nuclear export signal (NES) (aa79–99); the multimerization domains (MD) (aa57–66 and aa106–124), and the stability domain (SD) (aa170–189) [19,24]. Rex shuttles between the nucleus and the cytoplasm, transporting viral mRNA-encoding structural proteins to the site of translation and regulating virus particle formation [21,22].

On the other hand, eukaryotic cells are universally equipped with nonsense-mediated mRNA decay (NMD), which targets and degrades aberrant mRNAs with premature termination codons (PTCs) that exist more than 50–55 nt upstream of the exon junction complex (EJC). When the ribosome stops at the stop codon on the mRNA, the translation termination factors, eRF1 and eRF3, and the central regulators of NMD, Upf1 and SMG1, are recruited to the ribosome, forming the SURF complex. When the stop codon is located upstream of the EJC, the SURF complex and EJC form a decay inducing complex (DECID), which is recognized as an aberrant mRNA with PTCs. When DECID is formed, Upf1 is phosphorylated by SMG1, initiating the NMD pathway. Phosphorylated Upf1 and the aberrant mRNA are transferred to the processing body (p-body), where Upf1 dephosphorylation by SMG5/7 or SMG6 dissociates Upf1 from the mRNA and recruits nuclease, completing the degradation of the aberrant mRNA. Recently, it has been shown that the levels of normal mRNAs containing upstream open reading frame (uORF), programmed ribosomal frameshift (PRF) signal, and a long 3′-untranslated region (UTR) of more than 1000 nt are fine-tuned by NMD; thus, NMD is an essential mechanism for maintaining cellular homeostasis through the control of intracellular mRNA levels and quality [25,26,27].

It has been widely reported that viral RNA is a target of NMD, and RNA viruses have evolved their own strategies to protect viral RNA from NMD [28,29,30,31]. HTLV-1 encodes more than 10 different viral proteins within its 9 kbp proviral genome. This is made possible by multiple alternative splicing sites, sub-optimal AUGs, -1 ribosomal frameshift signal (-1RFS) and other RNA signals in the viral genome. We have previously shown that unspliced HTLV-1 mRNA is degraded as a target of NMD in human cells, while Rex has function to suppress NMD, stabilizes unspliced HTLV-1 mRNA and promotes translation of viral structural proteins [32]. It has also been reported that Tax inhibits NMD via interaction with Upf1 and eIF3E/INT6 [33,34]. Recently, Prochasson et al. elegantly reviewed the relationship between HTLV-1 and host NMD [35]. However, the offense and defense between HTLV-1 and NMD are complex and it remains unclear how HTLV-1 evades this powerful host mRNA quality control mechanism. We hypothesize that, in addition to Tax-mediated NMD repression, Rex also represses NMD during HTLV-1 mRNA trafficking, but its molecular mechanism remains unclear. In the present study, we aimed to identify the “NMD repressor domain” of Rex and to elucidate the mechanism of NMD repression by Rex by examining the interaction between Rex and host NMD regulatory proteins.

## 2. Materials and Methods

### 2.1. Cell Culture

CEM, Molt-4, Jurkat (T-ALL patient-derived T-cell lines), MT-2 (HTLV-1-immortalized T-cell lines), HeLa (cervical cancer-derived epithelial cell line), HEK293T (human embryonic kidney-derived cell line containing the SV40 T-antigen), and HEK293FT were used in the present study. All cell lines were obtained and maintained as previously reported [36]. About the origin of the cell lines, MT-2 was provided from Gunma University, Japan. Authentication was conducted in our laboratory using the proviral integration-site sequencing technique [37]. We also confirmed production of infectious HTLV-1 viral particles and expression of Gag-Tax fusion protein. Jurkat and HEK293FT were obtained from Riken Cell Bank, Japan. CEM, Molt-4, HeLa, and HEK293T cells were provided by the Japanese Foundation for Cancer Research (JFCR).

### 2.2. Construction of Protein Expression Plasmids in Mammalian Cells

#### 2.2.1. Construction of WT-Rex and Other Protein Expression Plasmids

The cDNAs were amplified by PCR with Platinum Taq High Fidelity (Invitrogen, Thermo Fisher Scientific, Inc., Waltham, MA, USA using the original plasmid containing the cDNAs encoding each protein as a template. The primers with appropriate restriction enzyme sites were used for PCRs. The amplicon after PCR was separated by agarose-gel electrophoresis, TA-ligated into pGEM-T-Easy vector (Promega, Corp., Madison, WI, USA), amplified in DH-5α *E. coli* strain, and purified by a miniprep kit (SIGMA-Aldrich, Merck KGaA, Darmstadt, Germany). After cleavage with restriction enzymes, the target cDNA was extracted by electrophoresis and ligated with the final vector. The sequence was confirmed before used in experiments. The list of template plasmids, primers (all are shown in 5′→ 3′) and final vectors used for plasmid preparation is shown below (Table 1).

#### 2.2.2. Construction of Mutant Protein Expression Plasmids

For the construction of expression plasmids of Rex mutants, Upf1 mutants and Rex(-)-pFL-MT-2, we used a primer set containing the desired deletion/substitution and introduced the mutations using the Prime STAR Mutagenesis Basal Kit (Takara Bio Inc., Shiga, Japan). The point mutants in the RNA-binding motif of Rex were adapted from those constructed by Rimsky et al. [41]. M1 and M2 were mutated in the ARM, and M3–5 were mutated in the aa20–56 region, respectively. M1 and M2 were shown to lose RNA-binding ability, and their localization was observed in the cytoplasm and in the nucleus/cytoplasm, whereas the RNA-binding activity of M3–5 was comparable to that of wild-type Rex. The Rex phosphorylation mutants are based on the comprehensive identification of phosphorylation sites in Rex by Kesic et al. [23]. The hyperphosphorylated mutant (G495R/G497E) [42] and the ATPase-deficient mutant (R843C) [43] of Upf1 were constructed based on previous reports. The template plasmids used to construct the mutant expression plasmids and the mutagenesis primers (all are shown in 5′→ 3′) are shown below (Table 2). In the present study, a HTLV-1 infectious clone, pFL-MT-2 [44], was used to establish the site of HTLV-1 genomic expression. To construct the Rex(-)-pFL-MT-2, the HTLV-1 infectious plasmid without Rex expression, the mutagenesis primers shown in Table 2 were used.

### 2.3. Establishment of Stable Rex-Expressing T-Cell Lines by Retroviral Expression

In order to establish T-cell lines that express Rex stably, we employed a retroviral expression system using pRx-Puro vector for Rex-expressing recombinant retrovirus preparation. After 24 h, HEK293FT cells were seeded on a 6 cm dish at 5 × 10^5^ cells/mL, 5 mL/dish, and 5 μg of gag/pol and 5 μg of env vector, 10 μg of pRx-Puro (Mock) or 10 μg of pRx-Puro-Rex were co-transfected by the calcium phosphate method. The culture medium was changed after 4 h and the supernatant h was filtered after 48 by a filter with a pore size of 0.45 μm and used as a virus solution. An amount of 1.0 × 10^6^ cells/1 mL/well of CEM or Molt4 were seeded on 6-well plates and 1 mL of the Mock or Rex-expressing retrovirus solution and 8 μg/mL of polybrene were added to infect the cells. After 48 h, puromycin selection (0.5 μg/mL) was started and the cells were used for experiments after 12 days.

### 2.4. Measurement of Cellular NMD Activity

#### 2.4.1. The β-Globin Luciferase Reporter Assay System

In this study, a highly sensitive and quantitative β-globin (WT/PTC) dual luciferase reporter system was used to measure the activity of NMD [32]. pCDNA6-RSV-Renilla-Luc-β-globin (WT) (Renilla-WT) is expressed independent of NMD activity in the cell, whereas pCDNA6-RSV-Firefly-Luc-β-globin (PTC) (Firefly-PTC), in which PTC is introduced into the second exon, is expressed dependent on NMD activity because its mRNA is targeted by NMD. NMD activity is therefore calculated as the ratio between Renilla-luciferase activity and Firefly-luciferase activity (Renilla-WT/Firefly-PTC). At 24 h after HeLa cells were seeded at 2 × 10^4^ cells/well, 6 wells each for one condition (i.e., *n* = 6) in 48-well plates, 100 ng each of Renilla-WT and Firefly-PTC, and 200 ng of various effector plasmids for a well were co-transfected by the calcium phosphate method. Then, the luciferase assay was performed after 24 h. Rex/Mock Molt-4 cells or Rex/Mock CEM cells were seeded at 2.5 × 10^5^ cells/well in 48-well plates, 6 wells each for one condition (i.e., *n* = 6), and co-transfected with 100 ng of Renilla-WT and 300 ng of Firefly-PTC for a well with Lipofectamine 2000 (Invitrogen, Thermo Fisher Scientific, Inc., Waltham, MA, USA). The luciferase assay was performed after 24 h. To examine the effect of the HTLV-1 infectious clone (pFL-MT2) on NMD activity, firstly sHeLa cells stably expressing Renilla-WT and Firefly-PTC were established. sHeLa cells were seeded at 2.0 × 10^4^ cells/well on 48-well plates well, 6 wells each for one condition (i.e., *n* = 6). After 24 h, 400 ng of pFL-MT2 or Rex(-)-pFL-MT-2 for a well was transfected using Lipofectamine 2000 (Invitrogen, Thermo Fisher Scientific, Inc., Waltham, MA, USA), and 48 h later, Luciferase assay was performed. The Luciferase assay was conducted using a dual-Luciferase reporter system (Promega, Corp., Madison, WI, USA) to measure the activity of Renilla-luciferase and Firefly-luciferase using a Centro LB 960 luminometer (Berthold Technologies GmbH & Co KG, Bad Wildbad, Germany).

#### 2.4.2. Measurement of NMD Activity Adapted for Flow Cytometry

β-globin(WT) and β-globin(PTC) cDNA fragments were subcloned into pmCherry-C1 or pEGFP-C1 (both from Clontech, Takara Bio, Inc., Shiga, Japan), respectively. These two reporter plasmids and pME-FLAG-Rex or pME-FLAG (5 μg each) were introduced into Jurkat (5 × 10^5^ cells) by electroporation (250 V, 1050 F, 720 Ω). After 24 h, EGFP-expressing cells and mCherry-expressing cells were detected by a flow cytometer (FACS Calibur, BD Biosciences, Franklin Lakes, NJ, USA) and the ratio of mCherry (WT)/EGFP (PTC) was calculated as relative NMD activity.

### 2.5. Inhibition of Rex Phosphorylation by H-7 (Protein Kinase C Inhibitor)

H-7 is a protein kinase C inhibitor and has been reported to inhibit the phosphorylation of Rex [45,46]. HeLa cells were seeded in 48 wells at 2 × 104 cells/250 L/well on 48-well plates After 24 h, the cells were co-transfected with 100 ng each of Renilla-WT and Firefly-PTC, and 200 ng of pME-FLAG (Mock) or pME-FLAG-Rex by Lipofectoamine2000 (Invitrogen, Thermo Fisher Scientific, Inc., Waltham, MA, USA). After 24 h, H-7 was added to a final concentration of 50 μM (20 h treatment). Similarly, H-7 was added at 40 h (4 h treatment), 42 h (2 h treatment), and at 44 h, and Luc assay was performed simultaneously with H-7-free (0 h treatment) samples.

### 2.6. Interaction between Rex and Host NMD Regulatory Complex Proteins

#### 2.6.1. Identification of NMD Regulatory Proteins Interacting with Rex

HEK293FT cells were seeded at a concentration of 5 × 10^5^ cells/mL and 10 mL/10 cm culture dish one day before transfection. At 24 h after seeding, 10 μg pMEG-2 or Rex-pMEG-2 plasmid was transfected by polyethylenimine (PEI). After 48 h, the cells were collected and whole-cell lysate was prepared in TBS buffer containing 1% NP-40. GST-Rex was then collected using Glutathione Sepharose 4G (Cytiva, Marlborough, MA, USA) and the coprecipitated NMD complex proteins were detected by Western blotting.

#### 2.6.2. Interaction between Upf2 and Upf3A or Upf3B with/without Rex

HEK293FT cells were seeded at a concentration of 5 × 10^5^ cells/mL and 10 mL/10 cm culture dish one day before transfection. At 24 h after seeding, 10 μg each of GST-Upf2, HA-UP3A, His-Upf3B with/without SRα-Rex plasmid was transfected by PEI. For the negative control, cells transfected with pMEG-2 (GST only) instead of GST-Upf2 were also prepared. After 48 h, the cells were collected and whole-cell lysate was prepared in TBS buffer containing 1% NP-40. After that, GST-Upf2 was collected using Glutathione Sepharose 4G (Cytiva, Marlborough, MA, USA), and the coprecipitated Upf3A and Upf3B were detected by Western blotting.

#### 2.6.3. Subcellular Interaction between Rex and Upf1 by FRET

In the present study, we employed fluorescence resonance energy transfer (FRET) analysis to identify the subcellular sites of Rex and Upf1 interaction. An energy transfer from donor (ECFP) to acceptor (EYFP) occurs when the distance between ECFP and EYFP is less than 10 nm, in which the two molecules are highly likely to be physically interacting.

We first prepared the expression plasmids of ECFP-Rex and EYFP-Upf1. HeLa cells were seeded at 1 × 10^5^ cells/mL, 200 μL/well, on a 4-well culture glass slide. After 24 h, both ECFP-Rex and EYFP-Upf1 expression plasmids were transfected to HeLa cells using Lipofectamine2000 (Invitrogen, Thermo Fisher Scientific, Inc., Waltham, MA, USA). After a further 24 h, cells were fixed in 4% paraformaldehyde, and sealed in the mounting medium containing DAPI in 80% glycerol. To eliminate the FRET signal derived from non-specific interactions other than Rex and Upf1, we prepared a series of negative-control HeLa cells expressing ECFP and EYFP; ECFP and EYFP-Upf1; ECFP-Rex and EYFP. To confirm that our FRET system indeed detects high efficiency FRET, we also prepared positive-control HeLa cells expressing tandemly connected ECFP + EYFP.

For high-efficiency FRET detection, we employed the acceptor-photobleaching using LSM710 confocal microscope (Carl Zeiss, AG., Oberkochen, Germany). Briefly, EYFP molecules in regions of interest (ROIs) of nuclei, cytoplasm, and p-bodies were photobleached by the laser of 541 nm at 100% power and 100-times iterations. The intensity of ECFP before and after photobleaching was measured every 1 s for 10 s, respectively, i.e., 10 measurements each before and after photobleaching. Calculation of FRET efficiency is as follows. FRET efficiency (%) = [1-(average of pre-photobleaching intensity of ECFP)/(average of post-photobleaching intensity of ECFP)] ×100. Higher FRET efficiency means closer positioning of ECFP and EYFP. Data were collected from 6 to 10 ROIs in one image (*n* = 6–10).

### 2.7. Western Blotting

Sample cells were suspended in RIPA buffer (10 mM Tris-HCl (pH 7.4), 1% NP-40, 0.1% sodium deoxycholate, 0.1% SDS, 0.15 M NaCl, 1 mM EDTA (pH 8.0)) with protein inhibitor cocktail (Nacalai Tesque, Inc., Kyoto, Japan) and PMSF. Protein assay reagent (Bio-Rad Laboratories, Inc., Hercules, CA, USA) was used to determine the protein concentration. The whole-cell lysate was diluted to 1 mg protein/mL in sample buffer and boiled for 5 min to obtain a sample. Proteins were then separated by SDS-PAGE using a 12.5% acrylamide gel and transferred to a PVDF membrane (MerckMillipore, Merck KGaA, Darmstadt, Germany) using a wet transfer device. The transferred membrane was immersed in blocking solution (5% skim milk in 0.1% Tween-20 containing Tris-buffered saline, TBST) and blocked for 1 h. After blocking, the membrane was immersed in the primary antibody solution 2000-fold diluted in blocking solution at 4 °C for 20 h. The membrane was then washed 3 times for 15 min in TBST. The membrane was then incubated with the secondary antibody solution diluted 2000-fold in blocking solution for 2 h at room temperature. Finally, the membrane was washed with TBST for 5 min x 3 times and the bands were detected in NBT/BCIP (Promega, Corp., Madison, WI, USA) solution. The primary and secondary antibodies used in this experiment were as follows.

Primary antibodies: Upf1(#9435S, Cell Signaling Technology, Inc. Danvers, MA, USA), Upf2 (#ab-28712-200, Abcam, Plc. Cambridge, UK), Upf3A (#H00065110-M06, Abnova, Corp.), Upf3B (#A303-688A, Bethyl Laboratories, Inc., Montgomery, TX, USA), SMG1 (#9149S, Cell Signaling Technology, Inc.), SMG5 (#ab-129107, Abcam, Plc.), SMG6 (#sc-50984-R, Santa Cruz Biotechnology, Inc., Dallas, TX, USA), SMG7 (#sc-134857, Santa Cruz Biotechnology, Inc., Dallas, TX, USA), β-Actin (#sc-69879, Santa Cruz Biotechnology, Inc., Dallas, TX, USA), FLAG (#F1804, SIGMA-Aldrich, Merck KgaA, Darmstadt, Germany), GST (#Cytiva 27-4577-01, SIGMA-Aldrich, Merck KgaA, Darmstadt, Germany), and monoclonal antibodies against HTLV-1 proteins (Tax, Rex, Gag(p53), and Env(gp24)) were generated by Yuetsu Tanaka at the University of Ryukyus.

Secondary antibodies: Alkaline phosphatase (AP)-conjugated anti-mouse IgG (#S3721), AP-conjugated anti-rabbit IgG (#S3731), AP-conjugated anti-rat IgG (#S383A), AP-conjugated anti-goat IgG (#V-1151) (all from Promega, Corp., Madison, WI, USA).

### 2.8. Statistical Analysis

Throughout the present study, two-tailed paired Student’s *t*-tests were performed to test the statistical difference between the experimental groups. Asterisks in the figures indicate a significant difference between the tested groups (*, *p* < 0.05; **, *p* < 0.01; and ***, *p* < 0.001, *n* > 3).

## 3. Results

### 3.1. NMD Inhibition by Rex at the Site of HTLV-1 Viral Genome Expression

#### 3.1.1. Rex Inhibits NMD in Human T Cells

**Figure 1:**
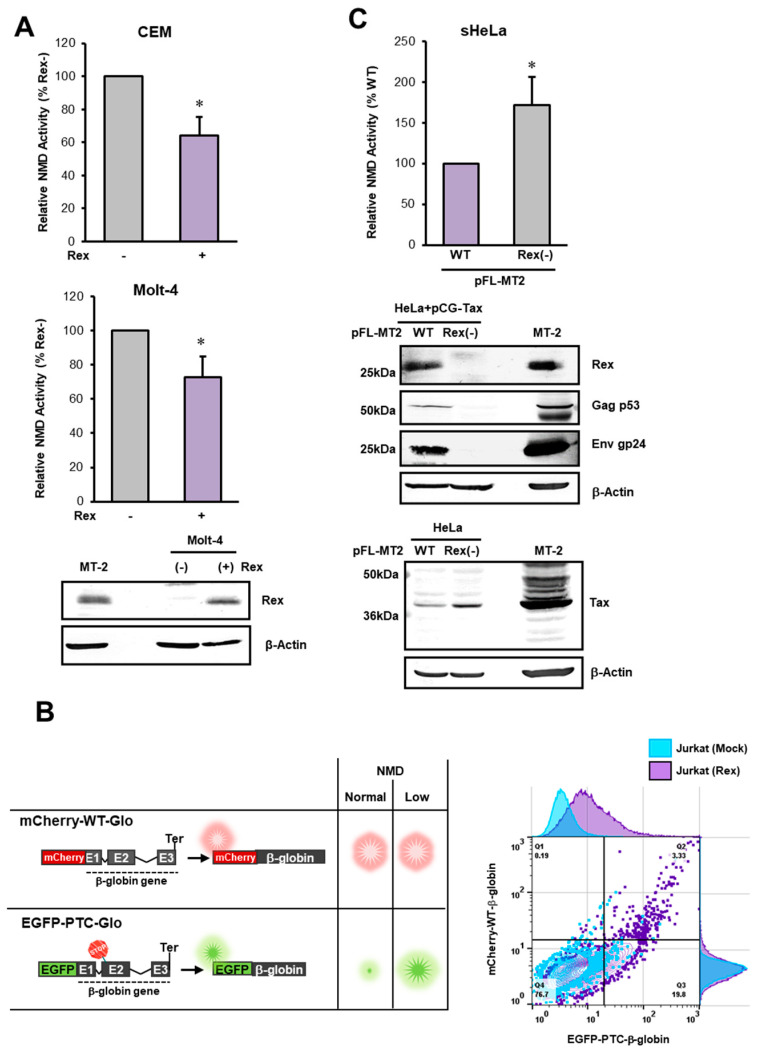
Rex inhibits NMD in T cells. (**A**). NMD activity was measured in CEM or Molt-4 cells constantly expressing Rex. The results showed a significant decrease in NMD activity in Rex-expressing cells of both cell lines (*n* = 6, mean ± SD, * *p* < 0.05). The image below shows Rex expression in Rex(+/-)-Molt-4 cells as detected by Western blotting. The whole-cell lysate of HTLV-1-infected immortalized cell line MT-2 is used as the positive control in Western blotting. (**B**). NMD-activity reporters (mCherry-WT (red) and EGFP-PTC (green)) were introduced into Jurkat, and the expression levels of mCherry and EGFP in the presence and absence of Rex were detected by flow cytometry (left figure). The results showed that the percentage of cells expressing EGFP increased in Rex-expressing cells compared to Mock cells (right panel), indicating that EGFP-β-globin (PTC) mRNA is stabilized in the presence of Rex. (**C**). Wild-type HTLV-1 infectious clone, pFL-MT2, or Rex-deficient clone, Rex(-)-pFL-MT2, were transfected into sHeLa cells stably expressing Renilla-WT and Firefly-PTC and evaluated for NMD activity. The results showed that NMD suppression was abolished in Rex (-) clone-transfected cells (top graph). In the cells with Rex(-)-pFL-MT2, the expression of Tax was upregulated and the expression of viral structural proteins such as Gag-p53 and Env-gp24 was markedly reduced (bottom image) (*n* = 6, mean ± SD, * *p* < 0.05).

We have previously reported that Rex inhibits NMD in HeLa cells [32], but not in human T cells, the site of HTLV-1 infection. In this experiment, we stably overexpressed Rex in CEM and Molt-4 T-cell lines using a retroviral expression system and analyzed the activity of NMD using the β-globin NMD reporter system. The results showed that NMD was significantly suppressed in Rex-expressing cells compared to control cells in both T cell lines (Figure 1A). The NMD activity in Jurkat cells with or without Rex was also analyzed by flow cytometry. The expression of EGFP-β-globin (PTC) was significantly increased in the presence in Rex, indicating that β-globin (PTC) mRNA was stabilized by the reduction in NMD activity (Figure 1B). These results indicate that host NMD is suppressed by Rex in T cells, which are the target of HTLV-1 infection.

#### 3.1.2. Effect of Rex on NMD Activity at the Site of HTLV-1 Viral Genome Expression

To clarify the effect of Rex on NMD in a more realistic HTLV-1 expression setting, a wild-type HTLV-1-infected clone, pFL-MT2 [44], and a Rex-deficient clone Rex(-)-pFL-MT-2 were transfected into sHeLa cells stably expressing the NMD reporter, and evaluated the NMD activity. The results showed that NMD was repressed in pFL-MT2 transfected cells, whereas NMD repression was abolished in Rex(-)-pFL-MT-2 transfected cells (Figure 1C). In Rex(-)-pFL-MT-2 transfected cells, the expression of Gag-p53 and Env-gp24 was decreased and that of Tax was increased in association with the loss of Rex expression (Figure 1C). These results indicate that the presence or absence of Rex directly affects host NMD activity at the site of HTLV-1 genome expression.

### 3.2. Identification of Rex Domains Important for NMD Suppression

**Figure 2:**
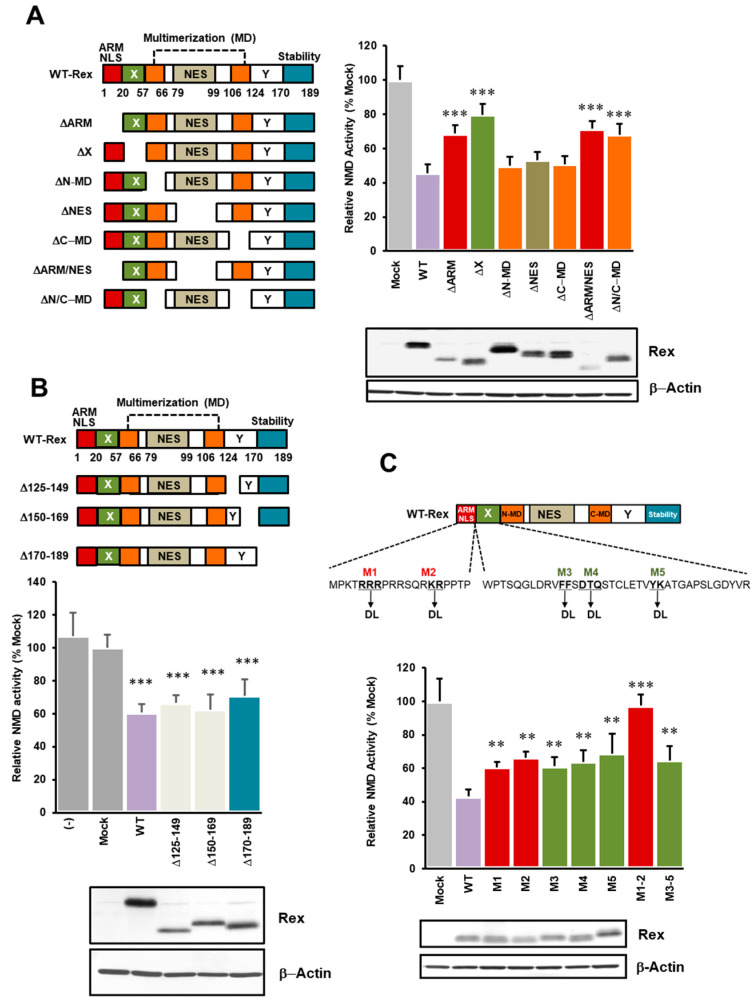
N-terminal region of Rex plays important roles in NMD inhibition. (**A**). We generated seven domain-deficient mutants of Rex (left panel) and compared the degree of NMD repression with WT-Rex using the NMD activity reporter assay system in HeLa cells. As shown in the graph on the right, NMD repression was significantly reduced when the ARM region, X region or both multimerization domains were deleted. Western blotting below shows the expression levels of each Rex mutant (*n* = 6, mean ± SD, *** *p* < 0.001, compared with WT-Rex). (**B**). For the C-terminal side, where the stabilizing domain of Rex is located, we also generated three types of deletion mutants (upper panel) and compared the degree of NMD repression with WT-Rex using the NMD activity reporter assay system in HeLa cells. As shown in the lower graph, there was no significant change in NMD repression in any of the mutants compared to WT-Rex. Western blotting below shows the expression levels of each Rex mutant (*n* = 6, mean ± SD, *** *p* < 0.001, compared with Mock). (**C**). We generated five point mutants in the ARM and X regions of Rex (upper panel) and compared the degree of NMD repression with WT-Rex using the NMD activity reporter assay system in HeLa cells. As shown in the lower graph, the degree of NMD suppression was significantly reduced in all mutants compared to WT-Rex. The reduction in NMD repression was particularly pronounced for both M1 and M2 mutants. Western blotting below shows the expression levels of each Rex mutant (*n* = 6, mean ± SD, ** *p* < 0.01; *** *p* < 0.001, compared with WT-Rex).

In this study, we compared the inhibitory effects of various deletion mutants of Rex on NMD and attempted to identify the Rex domain(s) that play an important role in NMD suppression. The results showed that the NMD repressive function of four Rex deletion mutants, ΔARM, ΔX (aa20–56), ΔARM/NES and ΔN/C-MD, was significantly reduced compared to wild-type (WT) Rex (Figure 2A). The C-terminal deletion mutants, Δaa125–149, Δaa150–169 and Δaa170–189, did not differ significantly in their ability to suppress NMD compared to WT-Rex (Figure 2B). Although it has been reported that this region contains a stability domain [24], the protein expression levels of these three mutants were similar to WT-Rex in the present study. Figure S1 shows that deletion of aa150–159 in the Y region resulted in almost no protein expression (mutant 3 and 4 compared with 2). Deletion of only the stability domain (aa170–189) (mutant 5) had no significant effect on protein expression, whereas deletion of aa160–169 with the stability domain markedly reduced protein expression (mutant 6 and 7 compared with 5, 9, and 10). These data indicate that part of the Y region plays an important for Rex stabilization. It was confirmed that there is no significant difference in the NMD inhibitory effect between mutant 10 and WT-Rex (Figure S1C). These results indicate that the ARM, an X region of unknown function (aa20–56), and at least one of the two multimerization domains are important for the function of Rex in NMD suppression.

### 3.3. ARM and X Region of Rex Play Important Roles in NMD Inhibition

Next, we used point mutants in the ARM and X regions (aa20–56) to elaborate on their relationship to the NMD repression mechanism: M1 (RRR5-7DL) and M2 (KR14-15DL) were mutated in the ARM region, M3 (FF30-31DL), M4 (DTQ33-35DL) and M5 (YK43-44DL) in the X region (Figure 2C). Comparison of the NMD repression of these mutants with that of WT-Rex showed that NMD activity was significantly restored in all mutants, confirming the importance of the ARM and X regions in NMD repression. The most significant recovery of NMD activity was observed when M1 and M2 of the ARM region were mutated simultaneously. Rimsky et al. [41] mentioned that M1 and M2 mutants lack the biological function of Rex to nuclear-export HTLV-1 Env mRNA and thus express Env protein, while M3, M4, and M5 maintained the Rex function. For Env expression, Rex needs to be transported to the nucleus and to bind to the RxRE of Env mRNA. The authors also showed that M1 localizes only in the cytoplasm, while M2 evenly localizes both in the nucleus and the cytoplasm. Our results show that M2, which can shuttle between the nucleus and the cytoplasm also lack the NMD suppressive activity. Therefore, the RNA-binding function of Rex may be related to its NMD-suppressive function.

### 3.4. Relationship between Rex Phosphorylation State and NMD Inhibition

**Figure 3:**
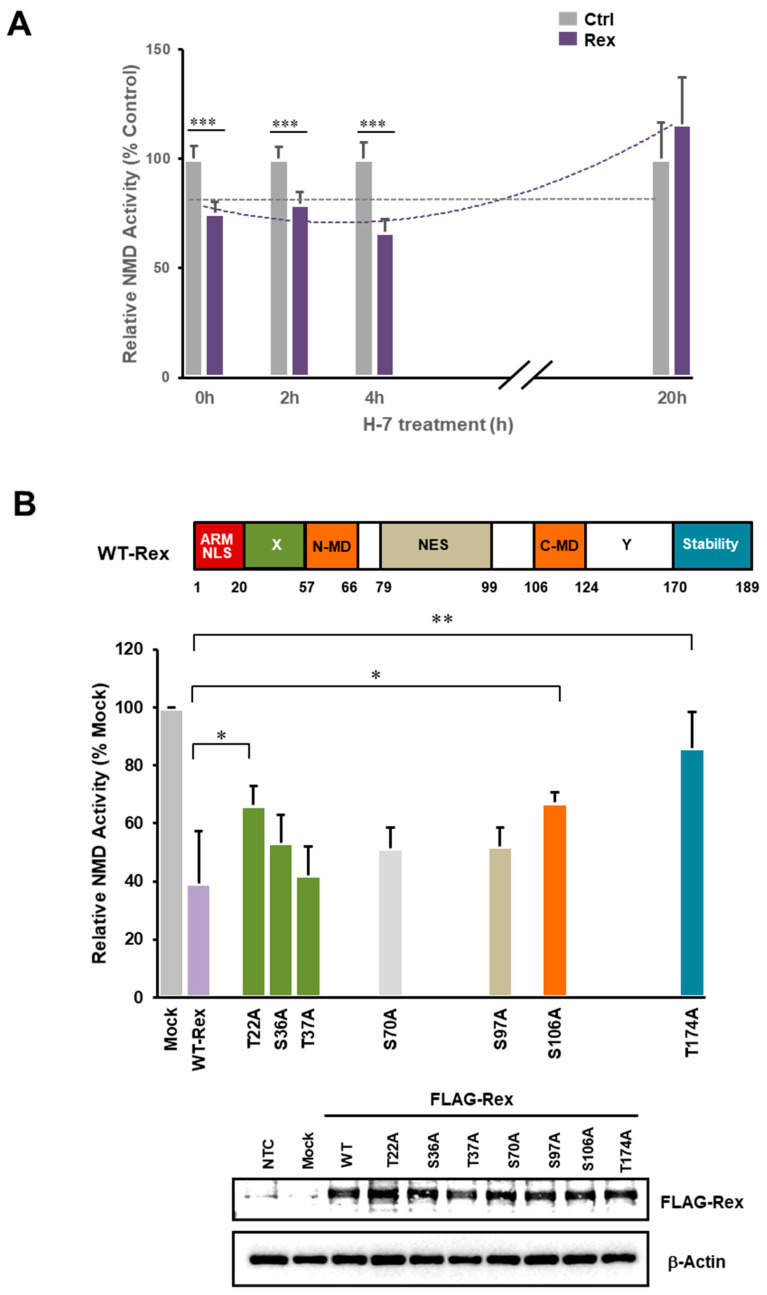
Phosphorylation status of Rex is related to its NMD inhibitory function. (**A**). Time course changes in the ability of Rex to inhibit NMD after treatment with the PKC inhibitor H-7 were examined using the NMD activity reporter assay system in HeLa cells. The results showed that Rex significantly inhibited NMD activity up to 4 h after H-7 treatment compared with the control but lost its ability to inhibit NMD after 20 h (*n* = 6, mean ± SD, *** *p* < 0.001). (**B**). Each mutant of seven phosphorylation sites of Rex was prepared and the NMD inhibitory ability was compared with that of WT-Rex using the NMD activity reporter assay system in HeLa cells. The results showed that the phosphorylation-deficient mutants at T22 in the X region, S106 in the C-terminal multimerization domain and T174 in the stability domain showed significantly reduced NMD repression. Western blotting below shows the expression levels of each Rex mutant (*n* = 6, mean ± SD, * *p* < 0.05; ** *p* < 0.01).

It is known that the activity of Rex is regulated by the phosphorylation status at multiple sites [23]. In the present study, H-7 (protein kinase C inhibitor) treatment abolished NMD inhibition (Figure 3A). Since H-7 treatment reduces the overall Rex phosphorylation to one-tenth of non-treated control [45], Figure 3A suggested that phosphorylation is also important to regulate the Rex activity in NMD inhibition. We then generated Rex phosphorylation mutants in which serine or threonine was replaced by alanine at seven phosphorylation sites [23], and compared the NMD-suppressive activity of these mutants with that of WT-Rex. We found that T22A, S106A and T174A mutants showed significantly reduced NMD repressive function (Figure 3B). These phosphorylation sites are located in the X region, the C-terminal multimerization domain and the stability domain, respectively, suggesting that phosphorylation in these regions may be important for NMD suppression.

### 3.5. Verification of CRM1 Dependence of NMD Suppression by Rex

**Figure 4:**
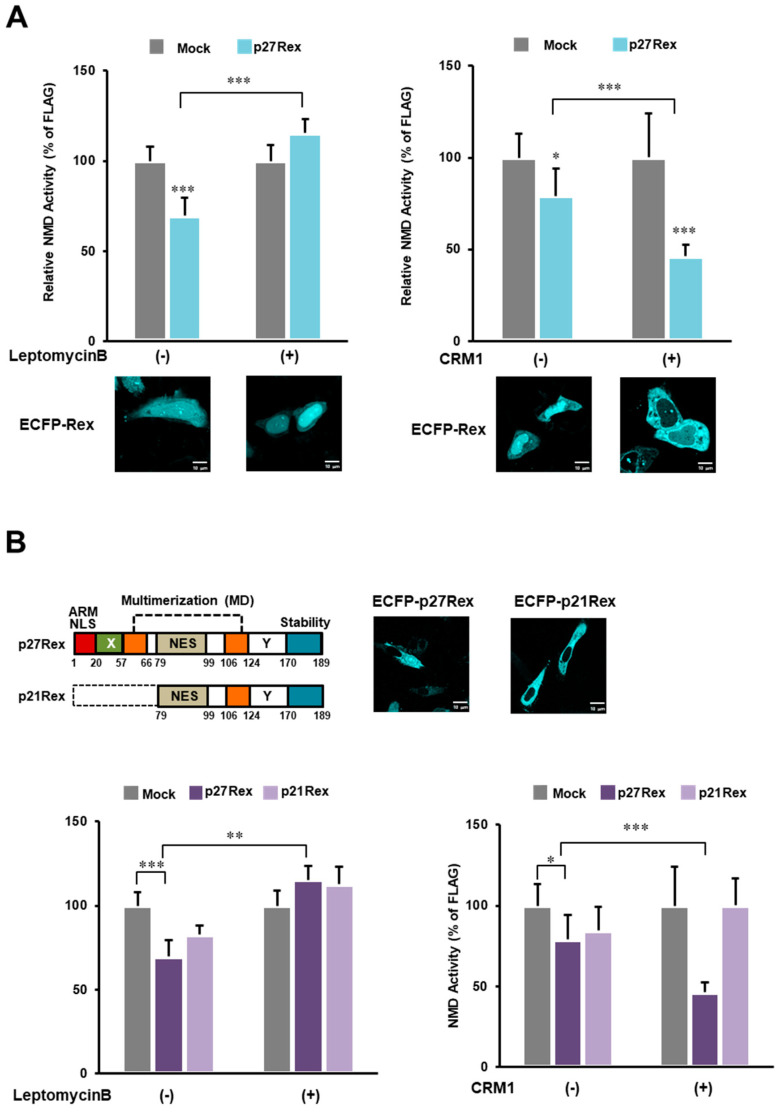
Rex needs to be nuclear-exported to inhibit NMD. (**A**). Treatment with Leptomycin B, an inhibitor of the nuclear export protein CRM1, significantly reduced the ability of Rex to inhibit NMD (left panel, *n* = 6, mean ± SD, *** *p* < 0.001). In contrast, overexpression of CRM1 significantly enhanced the ability of Rex to inhibit NMD (right panel, *n* = 6, mean ± SD, * *p* < 0.05; *** *p* < 0.001). The confocal laser microscopy images below show the subcellular localization of ECFP-Rex in HeLa cells subjected to each treatment. The scale bar indicates 10 μm (**B**). The data of the p21Rex mutant, which lacks NLS and thus localizes only in the cytoplasm (upper panel, The scale bar indicates 10 μm.), are added to the graphs of WT(p27)-Rex to compare the NMD-suppressive ability of WT(p27)-Rex with that of p21Rex under Leptomycin B treatment or CRM1 overexpression. Upon Leptomycin B treatment, only WT-Rex showed a significant decrease in NMD repression (left panel, *n* = 6, mean ± SD, ** *p* < 0.01; *** *p* < 0.001). When CRM1 was overexpressed, WT-Rex showed a significant enhancement of NMD repression, whereas p21Rex showed no change in NMD repression (right panel, *n* = 6, mean ± SD, * *p* < 0.05; *** *p* < 0.001).

It is known that Rex binds to the cellular nuclear export protein CRM1 and is transported to the cytoplasm [13,14]. In this study, we investigated how CRM1 inhibition by Leptomycin B treatment or CRM1 overexpression affects the ability of Rex to inhibit NMD. Leptomycin B treatment abolished the ability of Rex to inhibit NMD, whereas overexpression of CRM1 enhanced the ability of Rex to inhibit NMD (Figure 4A). This suggests that Rex exerts its NMD repressive function in the cytoplasm. This was also confirmed by experiments using p21Rex, a Rex mutant lacking N-terminal aa1–78 due to translation starting at the second ATG of the Rex ORF [47,48]. P21Rex lacks the NLS/ARM and therefore localizes to the cytoplasm. Because of its cytoplasmic localization, NMD activity in p21Rex-expressing cells was not affected by Leptomycin B treatment or CRM1 overexpression compared to p27(WT)Rex (Figure 4B). Thus, it appears that CRM1-dependent transport of Rex into the cytoplasm is required for Rex-mediated NMD repression.

### 3.6. Physiological Interaction between Rex and Upf1

**Figure 5:**
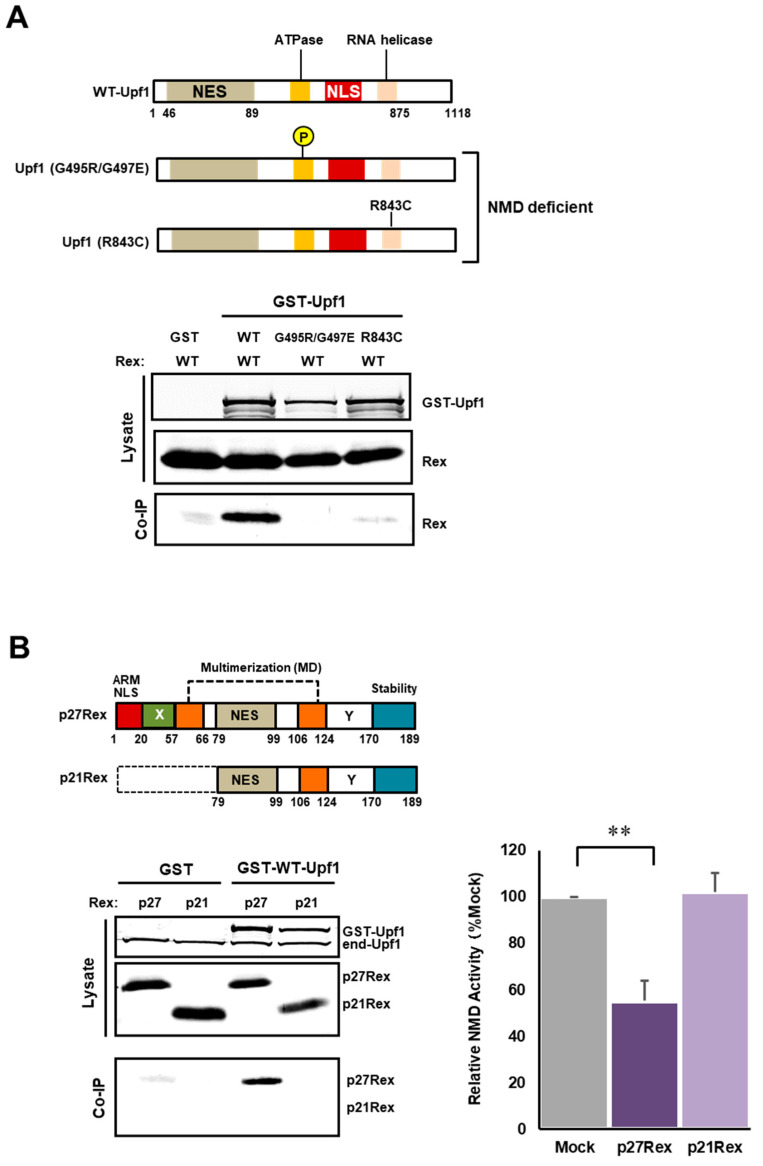
Only WT(p27)-Rex interacts with WT-Upf1. (**A**). Hyperphosphorylated and RNA helicase-deficient Upf1 mutants were generated (upper panel) and their interaction with WT-Rex was examined by GST pulldown assay in GST-Upf1 and FLAG-Rex-expressing HEK293FT cells. The results showed that Rex interacted only with WT-Upf1 and not with the NMD-deficient Upf1 mutants (lower panel). (**B**). The interaction of WT-Rex and p21Rex with WT-Upf1 was examined by GST pulldown assay in GST-Upf1 and FLAG-Rex-expressing HEK293FT cells. The results showed that only WT-Rex interacted with Upf1 (bottom left), which was correlated with NMD inhibitory capacity measured by the NMD activity reporter assay system in HeLa cells (bottom right) (*n* = 6, mean ± SD, ** *p* < 0.01).

Upf1 is a central regulator of NMD; phosphorylation and dephosphorylation of Upf1 are required for the activation and completion of the NMD pathway [49,50]. In addition, the ATPase activity of Upf1 is required for the completion of NMD and the disassembly of mRNPs [51]. We therefore generated NMD-deficient Upf1 mutants, a hyper-phosphorylation mutant Upf1 (G495R/G497E) [42] and an ATPase-deficient mutant Upf1 (R843C) [43], and examined their interaction with Rex. We found that Rex interacted only with WT-Upf1, but not with the NMD-activation-deficient Upf1 mutants (Figure 5A). Additionally, when the interaction of p27Rex and p21Rex with WT-Upf1 was compared, an interaction was observed only between p27Rex and WT-Upf1 (Figure 5B). As p21 Rex does not show NMD inhibition (Figure 5B, right panel graph), this suggests that the interaction of Rex with Upf1 via aa1–78, which contains the ARM and X regions, is required for NMD repression by Rex.

### 3.7. Subcellular Interaction between Rex and Upf1

**Figure 6:**
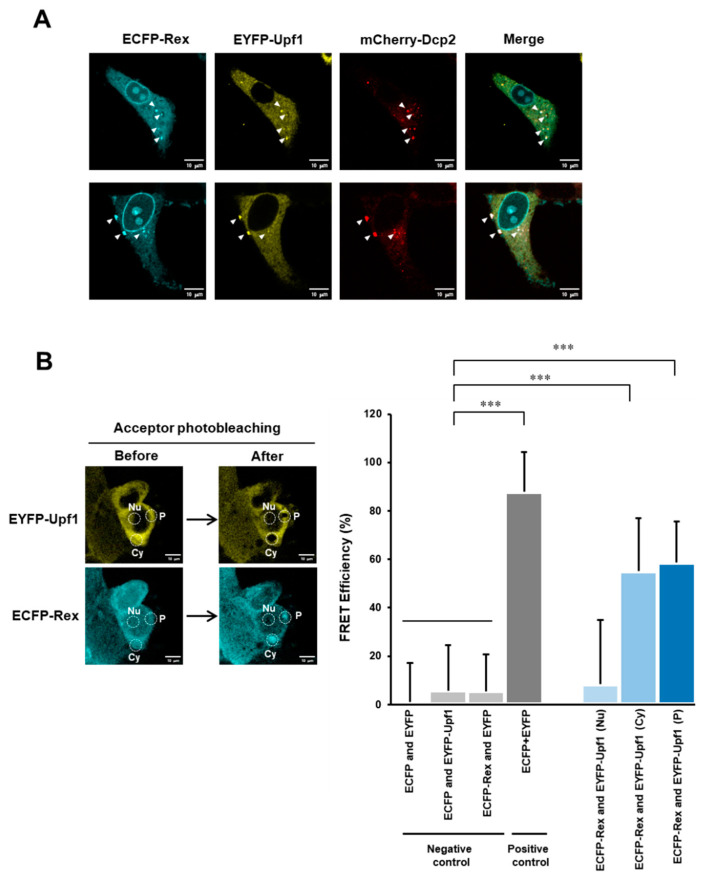
Rex interacts with Upf1 in the cytoplasm and in the p-body. (**A**). Confocal laser microscopy images of ECFP-Rex, EYFP-Upf1, and mCherry-Dcp2 in HeLa cells, where Dcp2 is a p-body marker. The white arrowhead indicates the site of the p-body. The scale bar indicates 10 μm. (**B**). The strength of the interaction between Rex and Upf1 in the cytoplasm, the p-body and the nucleus was analyzed by FRET. The left panel shows an example of images before and after acceptor photobleaching by confocal laser microscopy in HEK293FT cells co-expressing ECFP-Rex and EYFP-Upf1. The dotted white circles indicate regions where photobleaching was applied (Cy = cytoplasm, P = p-body, and Nu = nucleus, The scale bar indicates 10 μm.). The graph on the right shows the FRET efficiency (%). Significantly higher FRET efficiency was detected in the positive control (tandem ECFP + EYFP) than in the negative controls (ECFP and EYFP, ECFP and EYFP-Upf1, ECFP-Rex and EYFP), demonstrating that FRET efficiency is correctly measured in this method. The FRET efficiency between ECFP-Rex and EYFP-Upf1 was significantly higher in the cytoplasm and the p-body, indicating that Rex and Upf1 interact at these intracellular sites (*n* = 6–10, mean ± SD, *** *p* < 0.001).

When ECFP-Rex, EYFP-Upf1 and mCherry-Dcp2 were co-expressed in HeLa cells, Rex and Upf1 were shown to co-localize in the cytoplasm and the p-body (Figure 6A). We then calculated the FRET efficiency of ECFP-Rex and EYFP-Upf1 in these cells in the cytoplasm, the p-body and the nucleus. A significant increase in FRET efficiency was observed in the cytoplasm and the p-body (Figure 6B). These results indicate that Rex and Upf1 interact with each other in the cytoplasm and the p-body.

### 3.8. Interaction between Rex and NMD Regulatory Complex Proteins

**Figure 7:**
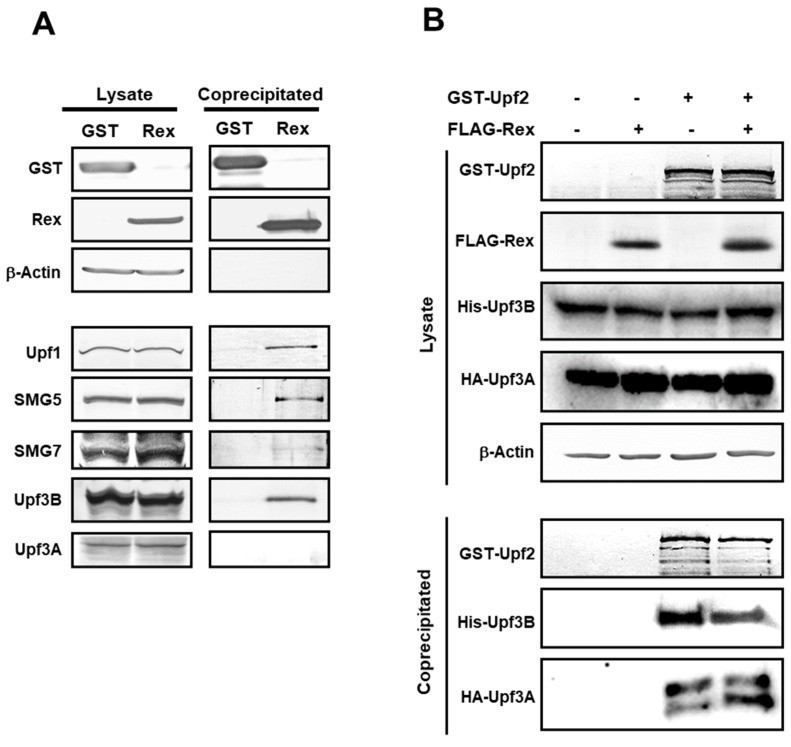
Rex interacts with Upf1 and SMG5/7 and contributes to the substitution of Upf3B and Upf3A. (**A**). NMD regulatory complex proteins interacting with Rex in HEK293FT cells were investigated by GST-Rex coimmunoprecipitation assay. The results showed that Rex interacts not only with Upf1 but also with SMG5, SMG7 and Upf3B. In contrast, Rex did not interact with Upf3A, the isoform of Upf3B. (**B**). Amount of Upf3B which binds to Upf2 in the NMD complex in the presence or absence of Rex detected by coimmunoprecipitation assay of GST-Upf2 with His-Upf3B and HA-Upf3A in HEK293FT cells. The amount of Upf3B interacting with Upf2 was reduced in the presence of Rex, and instead the interaction with Upf3A, the less active form of Upf3, was increased.

Finally, the interaction of Rex with NMD regulatory proteins was investigated by coimmunoprecipitation assay with GST-Rex. The results showed that Rex interacted with SMG5 and Upf3B as well as Upf1. It also interacted weakly with SMG7 (Figure 7A). We noted that Rex binds to Upf3B but not to Upf3A. The normal NMD complex contains Upf3B, which mediates the interaction between EJC and Upf1 via Upf2 when the NMD pathway is activated. On the other hand, it is known that when Upf3A is included in the NMD complex, NMD activity is reduced [52]. Therefore, we hypothesized that Rex selectively binds to Upf3B and inhibits its entry into the NMD complex, thereby suppressing NMD. We examined how the amounts of His-Upf3B and HA-Upf3A interacting with GST-Upf2 changed in the presence and absence of Rex by GST-Upf2 coimmunoprecipitation assay. As such, in the presence of Rex, the amount of Upf3B interacting with Upf2 decreased, whereas the amount of Upf3A increased (Figure 7B).

## 4. Discussion

Host cell NMD is a threat to the virus because many mRNAs derived from viral genomes have structures and RNA signals that are not found in eukaryotic cells and can be degraded via NMD as foreign substances. Previously, we have reported that Rex, an RNA-binding protein of HTLV-1, suppresses host NMD activity and stabilizes HTLV-1 genomic RNA [32]. However, the mechanism by which Rex achieves such a function has not been elucidated. In the present study, we “dissected” Rex to identify the important domain(s) of Rex for NMD repression by using various domain-deleted mutants of Rex. We also showed that Rex intervenes in the steps of NMD from the trigger to completion and may influence the function of multiple host factors that regulate NMD activity.

### 4.1. NMD Inhibition by Rex at the Mimiced Site of HTLV-1 Infection

In the present study, analysis of NMD activity in a stable Rex-expressing T cell line showed that Rex suppressed NMD in T cells, the host cells of HTLV-1 infection (Figure 1A,B). When the HTLV-1 infectious clone, pFL-MT2, was used to simulate a more realistic HTLV-1 infection, the activity of NMD was significantly increased in cells transfected with the Rex(-) clone compared with those transfected with the Rex(+) clone (Figure 1C). Because Rex represses Tax expression, Tax expression was increased in Rex(-)-pFL-MT-2 transfected cells compared with pFL-MT2 cells (Figure 1C, lower Western blotting image). It has been reported that Tax also represses NMD [33,34]. In our previous study, Tax was also found to inhibit NMD [32]. However, in the present study, NMD activity was significantly increased in cells transfected with Rex(-)-pFL-MT-2, even though Tax expression was upregulated. This suggests that Rex is mainly responsible for the NMD repressive function at the site where all HTLV-1 accessory proteins are expressed.

### 4.2. Identification of Rex Domains Important for NMD Suppression

The current study shows that ARM, the X region (aa20–56), whose function was previously unknown, or at least one of the two multimerization domains are important for the ability of Rex to suppress NMD (Figure 2A,C). In particular, loss of the X region was associated with a significant loss of NMD-suppressive activity, suggesting that the X region plays a central role in Rex-mediated NMD suppression (Figure 2A). Simultaneous loss of the two multimerization domains resulted in decreased NMD repression (Figure 2A), suggesting that multimerization is essential for Rex-mediated NMD repression. On the other hand, the Y region (aa124–169) and the stabilizing region (aa170–189) did not affect the expression level of Rex or its ability to suppress NMD (Figure 2B). As shown in Figure S1, deletion of aa150–159 in the Y region, as well as deletion of aa160–169 with the stability domain, markedly reduced protein. Therefore, the Y region may contain a motif which is important for stabilization of Rex protein.

### 4.3. Rex Phosphorylation State and NMD Inhibition

It is known that phosphorylation and multimerization of Rex are essential for the nuclear export and stabilization of unspliced and partially-spliced mRNAs of HTLV-1 [45,46]. We hypothesized that Rex phosphorylation is also required for NMD repression. We first inhibited the entire phosphorylation of Rex by H-7 and found that Rex-mediated NMD repression was abolished after 20 h of H-7 treatment (Figure 3A). This indicates that Rex phosphorylation is required for Rex-mediated NMD repression. Next, we used a series of Rex phosphorylation mutants to identify the phosphorylation sites of Rex that are important for NMD suppression. Of the seven phosphorylation sites in Rex, phosphorylation at T22, S106 and S174 was shown to be important for NMD inhibition (Figure 3B). T22 is located in the X region, S106 in the C-MD and S174 in the stability domain. The importance of the X region and MDs is consistent with the results using the domain-deficient mutant Rex proteins (Figure 2). Phosphorylation of S174 has also been reported to be essential for the activation of Rex [23]. The relationship between phosphorylation of T22 in the X region and its activation, which is important for NMD repression, needs to be further investigated.

### 4.4. Verification of CRM1 Dependence of NMD Suppression by Rex

To test the importance of nuclear export of Rex itself in NMD inhibition, we compared the ability of Rex to repress NMD after treatment with the CRM1 inhibitor Leptomycin B or overexpression of CRM1. We found that CRM1 inhibition reduced the ability of Rex to inhibit NMD, whereas overexpression of CRM1 enhanced the ability of Rex to inhibit NMD (Figure 4A). Since p21Rex, which lacks NLS and localizes only to the cytoplasm, had no such effect (Figure 4B), it is likely that CRM1-dependent transport of Rex to the cytoplasm is required for NMD repression. Since NMD functions in the cytoplasm in concert with translation [25], it is reasonable that NMD repression by Rex occurs in the cytoplasm.

In Figure 2A, however, ΔNES-Rex demonstrated the same NMD inhibitory activity as WT-Rex. Figure S2 shows that ΔNES-Rex is mainly in the nucleus but also in the cytoplasm at a lower level in HeLa cells. Therefore, we speculate that ΔNES-Rex in the cytoplasm exhibits NMD inhibitory activity, at least by ectopic overexpression.

### 4.5. Examination of Physical Interaction between Rex and NMD Factors

Finally, we investigated which interactions between Rex and NMD regulatory proteins are important for Rex-mediated NMD repression. We first examined the interaction between Rex and Upf1, the key regulator of NMD. The NMD cascade begins with phosphorylation of Upf1 by SMG1 and is completed by dephosphorylation by SMG5/7 [49,50]. Therefore, phosphorylation and dephosphorylation of Upf1 are essential for the execution of the NMD pathway. It has also been reported that the helicase activity of Upf1 is essential for the completion of NMD [51]. Previously, it has been reported that hyperphosphorylated Upf1 (G495R/G497E) and ATPase helicase-deficient Upf1 (R843C) are unable to activate NMD [42,43]. In the present study, we investigated the interaction between Rex and these Upf1 mutants by GST-Upf1 coimmunoprecipitation assay. The results showed that Rex interacted only with WT-Upf1 and hardly bound to mutant Upf1 (Figure 5A). It is possible that the helicase-deficient Upf1 mutant is not part of the active NMD complex and therefore has no opportunity to interact with Rex. It was also recently reported that hyper-phosphorylated Upf1 also plays an important role in the protein quality control machinery that transports C-terminal-deficient peptides, by-products of NMD target mRNAs, to the aggresome [53]. By not interacting with hyper-phosphorylated Upf1, Rex may avoid being transported to aggresome by Upf1.

When the interaction of WT-Upf1 with p27Rex or p21Rex was compared, only p27Rex interacted with Upf1 (Figure 5B). This result suggests that Rex interacts with Upf1 via the ARM/NLS and X regions of the N-terminal region, and that the interaction of p27Rex with Upf1 via the N-terminal region is important in NMD repression, as p21Rex did not show NMD repression (Figure 5B, right side graph). This result is in agreement with the results, which showed that the ARM and X regions of Rex are important for NMD repression (Figure 2 and Figure 3).

In addition, we analyzed in detail the intracellular interaction between Rex and Upf1. First, we co-expressed ECFP-Rex, EYFP-Upf1 and mCherry-Dcp2 in HeLa cells and investigated the subcellular localization of each protein. The results showed that Rex and Upf1 co-localized in the cytoplasm and the p-body as indicated by Dcp2 (Figure 6A). In addition, when the interaction between Rex and Upf1 was examined in the cell by FRET, the FRET efficiency of Rex and Upf1 was significantly increased in the cytoplasm and the p-body (Figure 6B). The p-body is a site of mRNA processing and mRNA degradation by the NMD pathway [54,55,56,57]. It has also been reported that viral mRNAs and proteins accumulate in the p-body [58]. Recently, it was also reported that p-bodies store translationally repressed mRNAs and regulate the efficiency of mRNA translation [59]. The initiation of the Upf1-centred NMD pathway occurs in the cytoplasm and is completed in the p-body. Therefore, Rex may interact with Upf1 from initiation to termination of NMD to regulate its activity.

Our GST-Rex immuno-coprecipitation assay shows that Rex interacts with Upf1, SMG5, Upf3B, and slightly with SMG7 (Figure 7A). SMG5 and SMG7 are responsible for dephosphorylation of Upf1, which is essential for completion of NMD [49,50]. Thus, Rex may inhibit NMD completion by suppressing the function of these proteins. Our results also showed that Rex interacted with Upf3B but did not interact with Upf3A (Figure 7A). The normal NMD complex mainly contains Upf3B interacting with Upf2. On the other hand, it has been reported that NMD activity decreases when Upf3A is included [52]. We investigated the possibility that Rex alters the amounts of Upf3B and Upf3A in the NMD complex. Our results demonstrated that, in the presence of Rex, the amount of Upf3B bound to Upf2 decreased and the amount of Upf3A increased (Figure 7B). These results suggest that Rex may contribute to the replacement of Upf3B and Upf3A in the NMD complex. We believe that Rex selectively binds to Upf3B, thereby preventing Upf3B from entering the NMD complex and allowing Upf3A to enter instead, thereby suppressing NMD activity.

### 4.6. Summary

**Figure 8:**
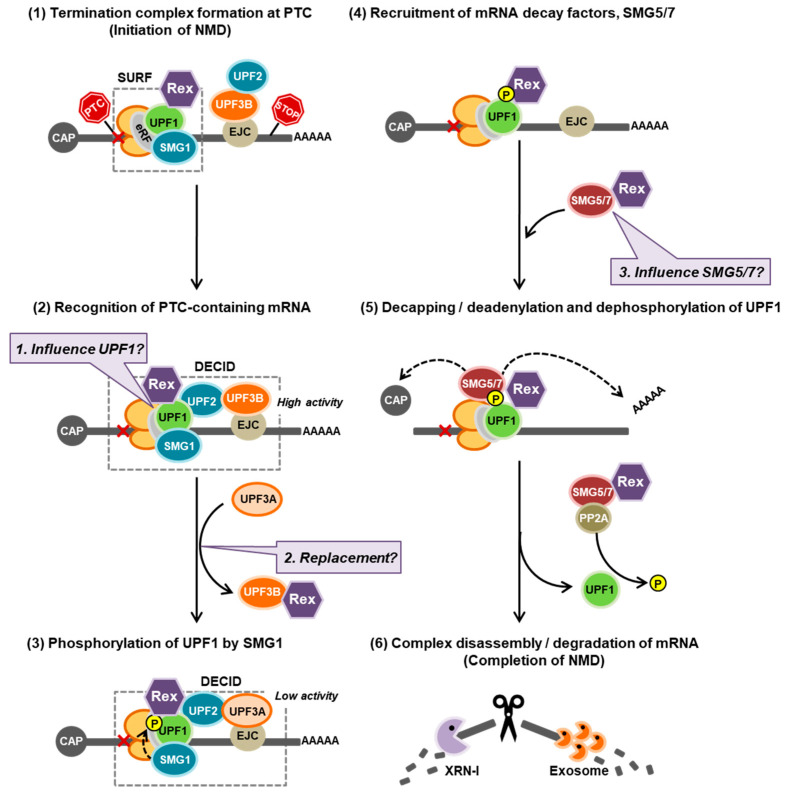
Possible model of Rex-NMD complex interaction and new questions. NMD progresses in a cascade as shown in (1–6). (**1**): Our results suggest that Rex may inhibit NMD by intervening from the beginning to the end of this cascade by interacting with the NMD key regulator Upf1 and may have some effect on its activity (Baloon-1). (**2**): Rex interacts only with Upf3B, suggesting that Rex substitutes Upf3A for Upf3B in the NMD complex, resulting in the inclusion of less active Upf3A in the NMD complex, thereby suppressing NMD activity (Baloon-2). (**4**,**5**): The mRNA decay factors, the SMG5/7 complex, are recruited to phosphorylated Upf1. The SMG5/7 complex recruits the decapping complex (DCP2/DCP1a) and the deadenylation complex CCR4–NOT to enhance decapping and deadenylation of the NMD target mRNA. Additionally, the SMG5/7 complex recruits protein phosphatase 2A (PP2A) for dephosphorylation of Upf1. Since dephosphorylation of Upf1 is essential for NMD completion, Rex may influence the dephosphorylation pathway of Upf1 via SMG5/7 interaction, and thus may influence NMD completion (Baloon-3).

In the current study, we found that the ARM region and the X region, whose function was previously unknown, at the N-terminus of Rex play important roles in NMD repression. This may be because Rex interacts with the NMD key regulator Upf1 via this region. Furthermore, the interaction between Rex and Upf1 was observed in the cytoplasm and p-bodies, where the NMD cascade functions. Indeed, Rex interacts not only with Upf1, but also with SMG5 and SMG7, which are essential for Upf1 dephosphorylation and NMD completion, suggesting that Rex may intervene in the NMD regulatory complex from initiation to termination of NMD. Finally, we found that Rex binds selectively to Upf3B, one of the two isoforms of Upf3, and that in the presence of Rex, the amount of Upf3B in the NMD complex is reduced and the intervention of Upf3A is promoted; since the intervention of Upf3A reduces NMD activity [52], it is possible that Rex may suppress NMD by swapping Upf3B and Upf3A in the complex. Thus, Rex may enter the NMD complex from the beginning to the end of NMD and may influence the cellular NMD activity (Figure 8). Such interaction between Rex and NMD regulatory proteins may occur on the HTLV-1 mRNA, because the HTLV-1 genomic mRNA that Rex transports out of the nucleus contains multiple RNA signals that activate NMD.

Protecting viral mRNA from host NMD is an essential function in viral replication. In this study, we proposed a new possibility that Rex may protect HTLV-1 genomic mRNA, a particularly vulnerable NMD target, from degradation by interacting with various NMD regulators. Rex may also regulate the timing of the initial active viral replication phase and the subsequent stable latent infection phase not only by the already-known function to transport viral mRNAs encoding structural proteins from the nucleus, but also by controlling the activity of NMD that targets these viral mRNAs.

## Figures and Tables

**Table 1:** Primers for constructions of protein expression plasmids.

Plasmid Name	Template	Primers	Subcloning to
His-CRM1	HeLa cDNA library	hCRM1(BamHI)-For: GGATCCATGCCAGCAATTATG hCRM1(XhoI)-Rev: CTCGAGTTAATCACACATTTC	pCDNA3.1C /his (Invitrogen, Thermo Fisher Scientific, Inc., Waltham, MA, USA)
GST-UPF1	UPF1 fragment was exerted from UPF1-pCDNA3.1/his C (Invitrogen, Thermo Fisher Scientific, Inc., Waltham, MA, USA) [32] and subcloned in pMEG-2 at EcoRI and XbaI.		
EYFP-UPF1	pEYFP-C1 (Clontech, Takara Bio Inc., Japan)	EYFP(HindIII)-For: CCCAAGCTTATCATGGTGAGCAAGG EYFP(BamHI)-Rev: CGGGGATCCCTTGTACAGCTCGTC	EYFP cDNA was subcloned in UPF1-pCDNA3.1/his C (Invitrogen, Thermo Fisher Scientific, Inc., Waltham, MA, USA) [32] at HindIII and BamHI.
ECFP-Rex	Rex-pME-FLAG [32]	P27Rex(HindIII)-For: CCCAAGCTTCTATGCCCAAGACCCGTCGG P27Rex(BamHI)-Rev: GCGGATCCCGGTCACATGGGGCAGGAG	pECFP-C1 (Clontech, Takara Bio Inc., Shiga, Japan)
DCP2-mCherry	DCP2 fragment was exerted from pMAG1-MN1(MBL Co., LTD, Japan)-DCP2 plasmid (Kind gift from Dr. Tadanori Yamochi) and subcloned to pmCherry-C1 (Clontech, Takara Bio Inc., Shiga, Japan) at BamH1.		
GST-UPF2	pMEG-2 [38]	GST(NotI)-For: GCGGCCGCCAATGTCCCCTATACTAGG GST+Stop(NheI)-Rev: GCTAGCTTATTTTGGAGGATGGTC	GST cDNA was subcloned to Rent2-GFP-pCDNA3.1 TOPO (gifted from Dr. Hal Dietz (Plasmid #17709; http://n2t.net/addgene:17709 (accessed on 20 December 2021); RRID: Addgene_17709, Addgene, Watertown, MA, USA) [39].
HA-UPF3A	UPF3A fragment was exerted from UPF3A-puc57 (Synthesized by GENEWIZ, South Plainfield, NJ, USA) and subcloned in pCDNA3-HA (Invitrogen, Thermo Fisher Scientific, Inc., Waltham, MA, USA) at NotI and XbaI.		
His-UPF3B	UPF3B fragment was exerted from UPF3B-puc57 (Synthesized by GENEWIZ, South Plainfield, NJ, USA) and subcloned in pCDNA3.1C/his (Invitrogen, Thermo Fisher Scientific, Inc., Waltham, MA, USA) at BamH1 and XhoI.		
GST-Rex	Rex-pME-FLAG	P27Rex(EcoRI)-For: GAATTCATGCCCAAGACCCG P27Rex(NotI)-Rev: GCGGCCGCTCACATGGGGCAGGAG	pMEG-2 [38]
Rex-pRxPuro	Rex-pME-FLAG	P27Rex(EcoRI)-For: GAATTCTCGCCACCATGCCCAAGACCCG P27Rex(NotI)-Rev: GCGGCCGCTCACATGGGGCAGGAG	pRx-Puro [40]

**Table 2:** Primers for mutagenesis.

Plasmid Name	Template	Primers for Mutagenesis
ΔARM-Rex	Rex-pME-FLAG	ΔARM-Rex-Top: CCTCGAGTGGCCCACTTCCCAGGGT ΔARM-Rex-Bottom: GTGGGCCACTCGAGGAATTCCTTGTC
Δaa20-56-Rex	Rex-pME-FLAG	Δaa20-56-Rex-Top: AACACCACCCGCCTACATCGTCACG Δaa20-56-Rex-Botom: TAGGCGGGTGGTGTTGGTGGTCTTTT
ΔN-MD-Rex	Rex-pME-FLAG	ΔN-MD-Rex-Top: TGTTCGGCCTGTCCAGAGCATCAGA ΔN-MD-Rex-Bottom: TGGACAGGCCGAACATAGTCCCCCAG
ΔNES-Rex	Rex-pME-FLAG	ΔNES-Rex-Top: CCCCATCGAGAGAACCTCTAAGACCC ΔNES-Rex-Bottom: GTTCTCTCGATGGGGTCCCAGGTGA
ΔC-MD-Rex	Rex-pME-FLAG	ΔC-MD-Rex-Top: AAGACCCTCCAGGCCATGCGCAAAT ΔC-MD-Rex-Bottom: GGCCTGGAGGGTCTTAGAGGTTCTCT
ΔARM/NES-Rex	ΔNES-Rex	ΔARM-Rex-Top: CCTCGAGTGGCCCACTTCCCAGGGT ΔARM-Rex-Bottom: GTGGGCCACTCGAGGAATTCCTTGTC
ΔN/C-MD-Rex	ΔC-MD-Rex	ΔN-MD-Rex-Top: TGTTCGGCCTGTCCAGAGCATCAGA ΔN-MD-Rex-Bottom: TGGACAGGCCGAACATAGTCCCCCAG
Δaa125-139-Rex	Rex-pME-FLAG	Rex(Δaa125-139)-Top: TCCTTCCAACCCACCCTTGGGCAGC Rex(Δaa125-139)-Bottom: GGTGGGTTGGAAGGAGGGTGGAATGT
Δaa125-149-Rex	Rex-pME-FLAG	Rex(Δaa125-149)-Top: TCCTTCCTGTCTTTTCCAGACCCCG Rex(Δaa125-149)-Bottom: AAAAGACAGGAAGGAGGGTGGAATGT
Δaa125-159-Rex	Rex-pME-FLAG	Rex(Δaa125-159)-Top: TCCTTCCAAAACCTGTACACCCTCT Rex(Δaa125-159)-Bottom: CAGGTTTTGGAAGGAGGGTGGAATGT
Δaa125-169-Rex	Rex-pME-FLAG	Rex(Δaa125-169) -Top: CTCCTTCCTTGTCTGCATGTACCTCT Rex(Δaa125-169)-Bottom: CAGACAAGGAAGGAGGGTGGAATGT
Δaa170-189-Rex	Rex-pME-FLAG	Rex(Δaa170-189)-Top: AGGCTCCGTGAACTAGTCTAGAGAAA Rex(Δaa170-189)-Bottom: TAGTTCACGGAGCCTCCCCAGAGGG
Δaa160-189-Rex	Rex-pME-FLAG	Rex(Δaa160-189)-Top: CCGGCCCCTGAACTAGTCTAGAGAAA Rex(Δaa160-189)-Bottom: TAGTTCAGGGGCCGGAGTCCGGGGT
Δaa150-189-Rex	Rex-pME-FLAG	Rex(Δaa150-189)-Top: CCCAACCCTGAACTAGTCTAGAGAAA Rex(Δaa150-189)-Bottom: TAGTTCAGGGTTGGGAGGTGCTGCC
Δaa150-159-Rex	Rex-pME-FLAG	Rex(Δaa150-159)-Top: CCAACCCAAAACCTGTACACCCTCT Rex(Δaa150-159)-Bottom: CAGGTTTTGGGTTGGGAGGTGCTGCC
Δaa160-169-Rex	Rex-pME-FLAG	Rex(Δaa160-169)-Top: CGGCCCCTTGTCTGCATGTACCTCT Rex(Δaa160-169)-Bottom: GCAGACAAGGGGCCGGAGTCCGGGGT
Δaa150-169-Rex	Rex-pME-FLAG	Rex(Δaa150-169)-Top: CCAACCCTTGTCTGCATGTACCTCT Rex(Δaa150-169)-Bottom: GCAGACAAGGGTTGGGAGGTGCTGCC
Rex-M1 (RRR5/6/7DL)	Rex-pME-FLAG	Rex(M1) -Top: AGACCGATCTGCCCCGCCGATCCCAAAG Rex(M1)-Bottom: GGGGCAGATCGGTCTTGGGCATCTCGAG
Rex-M2 (KR14/15DL)	Rex-pME-FLAG	Rex(M2)-Top: AAAGAGATCTACCACCAACACCATGGCC Rex(M2)-Bottom: GTGGTAGATCTCTTTGGGATCGGCGGGG
Rex-M3 (FF30/31DL)	Rex-pME-FLAG	Rex(M3)-Top: GAGTCGATCTTTCGGATACCCAGTCTAC Rex(M3)-Bottom: CCGAAAGATCGACTCTGTCCAAACCCTG
Rex-M4 (DTQ33/34/35DL)	Rex-pME-FLAG	Rex(M4)-Top: TTTCGGATCTGTCTACGTGTTTGGAGAC Rex(M4)-Bottom: TAGACAGATCCGAAAAGAAGACTCTGTC
Rex-M5 (YK43/44DL)	Rex-pME-FLAG	Rex(M5)-Top: CTGTGGATCTGGCGACTGGTGCCCCATC Rex(M5)-Bottom: TCGCCAGATCCACAGTCTCCAAACACGT
Rex (T22A)	Rex-pME-FLAG	Rex (T22A)-Top: CCAACACCATGGCCCGCTTCCCAGGGTTTGG Rex (T22A)-Bottom: CCAAACCCTGGGAAGCGGGCCATGGTGTTGG
Rex (S36A)	Rex-pME-FLAG	Rex (S36A)-Top: TTTTCGGATACCCAGGCTACGTGTTTGGAGA Rex (S36A)-Bottom: TCTCCAAACACGTAGCCTGGGTATCCGAAAA
Rex (T37A)	Rex-pME-FLAG	Rex (T37A)-Top: TCGGATACCCAGTCTGCGTGTTTGGAGACTG Rex (T37A)-Bottom: CAGTCTCCAAACACGCAGACTGGGTATCCGA
Rex (S70A)	Rex-pME-FLAG	Rex (S70A)-Top: TGGCCACCTGTCCAGGCCATCAGATCACCTGG Rex (S70A)-Bottom: CCAGGTGATCTGATGGCCTGGACAGGTGGCCA
Rex (S97A)	Rex-pME-FLAG	Rex (S97A)-Top: CTCGACTCCCCTCCTGCCCCACCCAGAGAAC Rex (S97A)-Bottom: GTTCTCTGGGTGGGGCAGGAGGGGAGTCGAG
Rex (S106A)	Rex-pME-FLAG	Rex (S106A)-Top: GAACCTCTAAGACCCGCAAGGTCCTTACCCC Rex (S106A)-Bottom: GGGGTAAGGACCTTGCGGGTCTTAGAGGTTC
Rex (T174A)	Rex-pME-FLAG	Rex (T174A)-Top: CCGTTGTCTGCATGTGCCTCTACCAGCTTTC Rex (T174A)-Bottom: GAAAGCTGGTAGAGGCACATGCAGACAACGG
GST- UPF1(G495R/G497E)	GST-UPF1	hUPF1(G495R/G497E)-Top: AGGGCCCGCCAAGAACGGAGAAGACGGTGAC hUPF1(G495R/G497E)-Bottom: TCACCGTCTTCTCCGTTCTTGGCGGGCCCT
GST- UPF1(R843C)	GST-UPF1	hUPF1(R843C)-Top: TCCTGTCCTGTGTGTGTGCCAACGAGCACCA hUPF1(R843C)-Bottom: TGGTGCTCGTTGGCACACACACAGGACAGGA
Rex(-)-pFL-MT2	pFL-MT2 [44]	p27Rex(ATG-to-CTG)-Top: CCTCAAGCGAGCTGCCTGCCCAAGACCCGTC p27Rex(ATG-to-CTG)-Bottom: GACGGGTCTTGGGCAGGCAGCTCGCTTGAGG

## Data Availability

Not applicable (No public data was used in this study. Data in this study are not available in public.)

## Supplementary Materials

The following supporting information can be downloaded at: https://www.mdpi.com/article/10.3390/v14020344/s1, Figure S1: Y-region contains a motif important for stabilization of Rex; Figure S2: Subcellular localization of FLAG-Rex in HeLa cells.
