# Supplementary material for: Elucidation of the Mechanism of Host NMD Suppression by HTLV-1 Rex: Dissection of Rex to Identify the NMD Inhibitory Domain
Source: Viruses. 2022 Feb 9;14(2):344. doi: 10.3390/v14020344 (PMC8875924; doi:10.3390/v14020344)
Supplement: Supplementary file 1 [file viruses-14-00344-s001.zip › viruses-1551956-supplementary.pdf]

Supplementary data
Nakano et al., Elucidation of the mechanism of host NMD suppression by HTLV-1 Rex: Dissection of Rex to identify “NMD inhibitory domain”

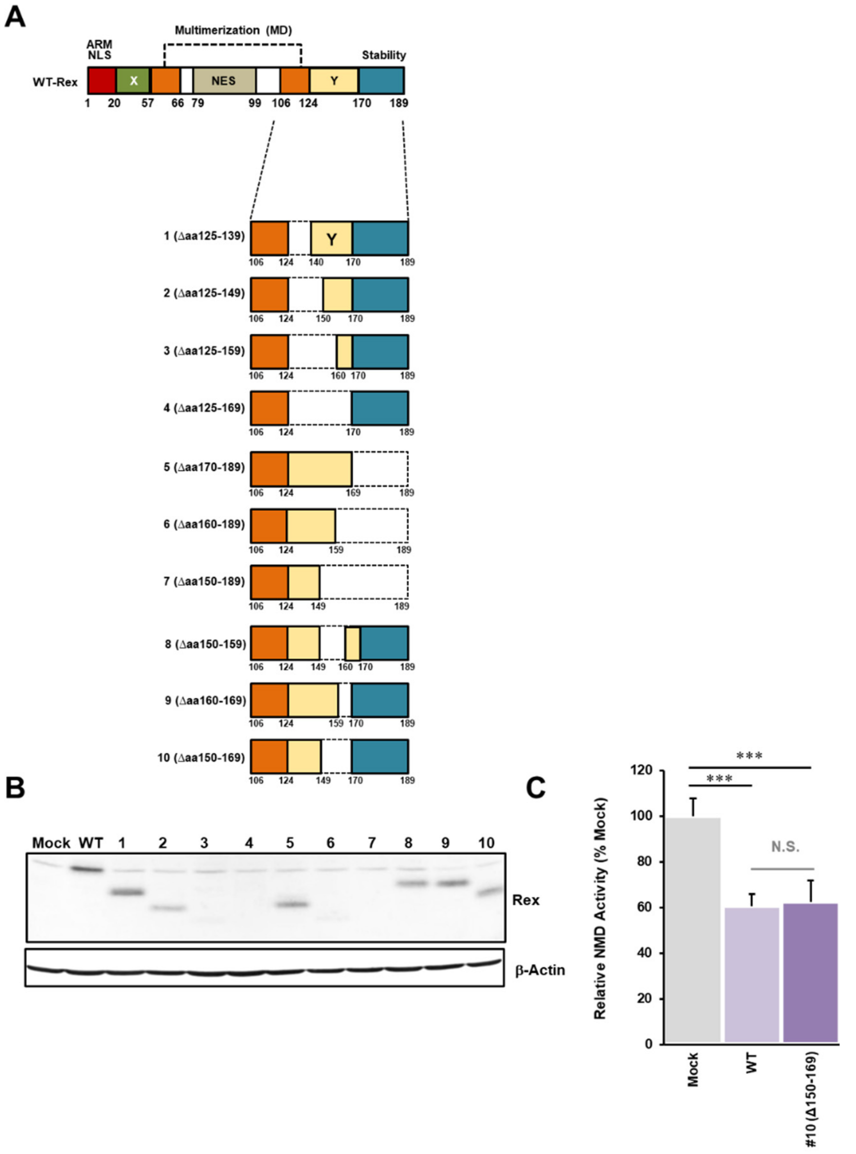

Figure S1. Y-region contains a motif important for stabilization of Rex.
A. Ten Rex deletion mutants in Y-region, of which function has not been clarified by using the 10 deletion mutants of Rex as shown. **B.** Expression levels of those 10 deletion mutants were compared with WT-Rex by Western blotting. Deletion of aa150-159 in the Y-region resulted in almost no protein expression (mutant-#3 and #4 compared with #2). Deletion of only the stability domain (aa170-189) (mutant-#5) had no significant effect on protein expression, whereas deletion of aa160-169 with the stability domain markedly reduced protein expression (mutant-#6 and #7 compared with #5, #9, #10). **C.** The NMD activity was compared between HeLa cells expressing WT-Rex or mutant-Rex-#10 (Δaa150-169). The graph shows that the mutant-#10 suppresses the NMD activity of the cells to the same extend as WT-Rex comparing with Mock cells. (n=6, Mean±SD, ***$p<0.001$)

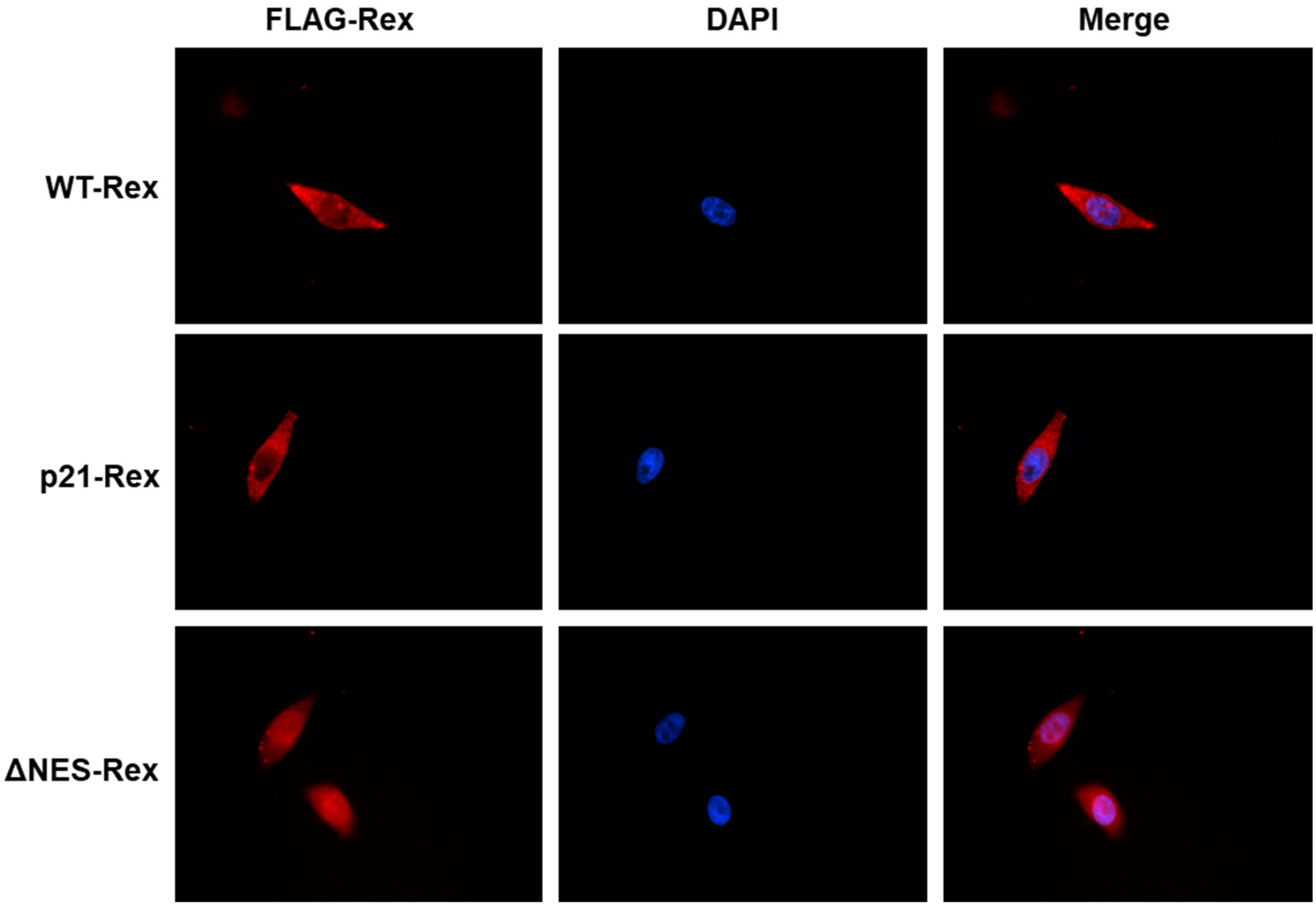

Figure S2. Subcellular localization of FLAG-Rex in HeLa cells.
FLAG-tagged WT-Rex, P21Rex, and ΔNES-Rex were overexpressed in HeLa cells and the subcellular localization was examined by immunocytochemistry with anti-FLAG antibody. WT-Rex distributes both in nucleus and cytoplasm, while p21-Rex without NLS region is only in cytoplasm, as already reported. ΔNES-Rex is mainly in nucleus but also in cytoplasm in a lower level.
